# Theory of mind, emotion recognition, delusions and the quality of the therapeutic relationship in patients with psychosis – a secondary analysis of a randomized-controlled therapy trial

**DOI:** 10.1186/s12888-020-2482-z

**Published:** 2020-02-10

**Authors:** Stephanie Mehl, Klaus Hesse, Anna-Christine Schmidt, Martin W. Landsberg, Daniel Soll, Andreas Bechdolf, Jutta Herrlich, Tilo Kircher, Stefan Klingberg, Bernhard W. Müller, Georg Wiedemann, Andreas Wittorf, Wolfgang Wölwer, Michael Wagner

**Affiliations:** 1 Department of Psychiatry and Psychotherapy, University of Bonn, Sigmund-Freud-Straße 25, 53105 Bonn, Germany; 2 Department of Psychiatry and Psychotherapy, Philipps-University of Marburg, Rudolf-Bultmann-Straße 8, 35039 Marburg, Germany; 3 Department of Psychiatry and Psychotherapy, University of Tübingen, Calwerstraße 14, 72076 Tübingen, Germany; 4 Department of Psychiatry and Psychotherapy, University of Cologne, Gleuler Straße, 50931 Köln, Germany; 5 Department of Psychiatry and Psychotherapy, Vivantes Hospital Berlin, Dieffenbachstraße 1, 10967 Berlin, Germany; 6 Department of Psychiatry, Psychosomatic and Psychotherapy, University of Frankfurt, Heinrich-Hoffmann-Straße 10, 60528 Frankfurt am Main, Germany; 7 Department of Psychiatry and Psychotherapy, University of Duisburg- Essen, Virchowstraße 147, 45147 Essen, Germany; 8 Department of Psychiatry and Psychotherapy, Hospital Fulda, Pacelliallee 4, 36043 Fulda, Germany; 9 Department of Psychiatry and Psychotherapy, Medical Faculty, Heinrich-Heine University of Düsseldorf, Bergische Landstraße 2, 40629 Düsseldorf, Germany

**Keywords:** Schizophrenia, Delusions, Theory of mind, Emotion recognition, Quality of the therapeutic relationship, Interactional problems

## Abstract

**Background:**

Cognitive models of psychosis postulate an important role of Theory of mind (ToM) in the formation and maintenance of delusions, but research on this plausible conjecture has gathered conflicting findings. In addition, it is still an open question whether problems in emotion recognition (ER) are associated with delusions. We examined the association of problems in ToM and ER with different aspects of delusions in a large sample of patients with psychosis enrolled in a therapy trial. This also enabled us to explore the possible impact of ToM and ER on one part of patients’ social life: the quality of their therapeutic relationship.

**Methods:**

Patients with psychotic disorders and delusions and/or hallucinations (*n* = 185) and healthy controls (*n* = 48) completed a ToM picture sequencing task and an ER task. Subsequently, patients were enrolled in a randomized-controlled Cognitive Behavior Therapy (CBT) trial (ISRCTN29242879). Patients and therapists rated the quality of the therapeutic relationship during the first five sessions of therapy.

**Results:**

In comparison to controls, patients were impaired in both ToM and ER. Patients with deficits in ER experienced more severe delusional distress, whereas ToM problems were not related to delusions. In addition, deficits in ER predicted a less favorable therapeutic relationship and interactional problems viewed by the therapist. Impaired ER also moderated (increased) the negative influence of delusions on the therapeutic relationship and interactional difficulties viewed by the therapist.

**Conclusions:**

Cognitive models on the formation and maintenance of delusions should consider ER as a potential candidate that might be related to the formation and maintenance of delusional distress, whereas problems in ToM might not be directly related to delusions and secondary dimensions of delusions. In addition, problems in ER in patients with psychosis might have an impact on the quality of the therapeutic relationship and patients with problems in ER are more likely to be viewed as problematic by their therapists. Nevertheless, training ER might be a way to improve the quality of the therapeutic relationship and potentially the effectiveness of CBT or other interventions for patients with psychosis.

## Background

Theory of Mind (ToM) is defined as the ability to attribute other peoples’ mental states, intentions and emotions and to understand and predict their behaviour [1]. It is part of the concept of social cognition, i.e. the perception, processing and interpretation of social signals [2, 3]. Frith [4] proposed that deficits in ToM are a predisposing factor for persecutory delusions. Based on this assumption, ToM in patients with psychosis was found to be more than one standard deviation below the performance of healthy controls [5, 6]. In addition, several studies found evidence of an association between ToM deficits and persecutory [7] and general delusions [8]. Consequently, ToM has been incorporated into theoretical models as one causal factor involved in the formation and maintenance of delusions [9, 10] and positive symptoms of psychosis [11].

Nevertheless, in a recent review, ToM problems were associated with more severe general delusions or delusions of persecution in about half of all studies that addressed this question, whereas the other studies did not find an association [12]. Consequently, several newer theoretical models excluded ToM problems as a causal factor for persecutory delusions or positive symptoms [12, 13].

One explanation for the inconsistent results could be the small sample size of most studies investigating associations between ToM and delusions that was also not mended by the latest meta-analysis: rather than assessing the relation between ToM and delusions the authors investigated the relationship of ToM and reality distortion (an aggregation of delusions and hallucinations) and did not find any evidence for an association [14].

In addition, it is possible that problems in ToM are rather a risk factor for psychosis in general than delusions, whereas more basal problems of patients with psychosis in emotion recognition (ER), the ability to identify other persons’ emotions by using emotionally salient information in the environment (verbal and non-verbal cues) [15] might be more important in the formation and maintenance of delusions. This is suggested by the fact that patients with psychosis are severely impaired in ER in comparison to controls [16, 17] and by the results of the meta-analysis mentioned above that suggest an association between problems in ER and reality distortion [14, 18].

Further, as other social-cognitive biases as the jumping to conclusions-bias are related with more severe delusional frequency and delusional distress [19], it is possible that problems in ToM and ER problems might also enhance the frequency and distress caused by delusions, this question has not been addressed until today.

While an associations with delusions is unclear, ToM problems in patients with psychosis are closely related to problems with their social performance such as inadequate eye contact, speech modulation and conversational flow [20], interpersonal difficulties [21, 22] and problems in social functioning [23, 24]. Nevertheless, it is unclear whether problems in ToM and ER in patients with psychosis also affect the quality of their social interactions in real life.

One paradigm of a social interaction in real life is the therapeutic alliance defined as affective bond and consensus with regard to goals and treatment tasks [25]. As the therapeutic alliance is highly important for the effectiveness of Cognitive Behavior Therapy (CBT [26–28]), it is crucial to assess whether problems in ToM and ER might have a negative effect on it. This question has been investigated in one study reporting associations between ToM problems and patients’ ratings on the quality of the therapeutic alliance [29]. With regard to problems in ER, their impact on the therapeutic alliance has not been addressed until today, but an impact might be possible. If either ToM problems and/or deficits in ER might be associated with the therapeutic relationship, Cognitive Behavior Therapy (CBT) should more strongly address patients’ problems in ToM and ER to improve the therapeutic alliance and consequently its effectiveness.

Besides TOM and ER abilities, persecutory delusions also negatively impact the therapeutic alliance in CBT [30]. Interestingly, preserved ToM abilities were found to moderate (reduce) the negative influence of persecutory delusions on social functioning [31]. Thus, it is likely that positive ToM and ER performance might protect patients from the additional negative impact of delusions on the quality of the therapeutic relationship. Patients with preserved ToM and ER performance might be more able to understand the intentions and emotions of their therapists correctly and act accordingly, while still assuming that other persons are to be mistrusted. But, the question whether intact ToM and ER performance might reduce (moderate) the negative impact of delusions on the therapeutic relationship has not been assessed until today.

The present study was a secondary analysis of a randomised-controlled therapy trial [32] and was set out to investigate whether problems in ToM and ER are more severe in patients with psychosis (in comparison to controls (hypothesis 1)) and whether they are related to delusions and secondary dimensions of delusions (frequency and distress) in a large patient sample (hypothesis 2). Both hypothesis 1 and 2 have been pre-specified in the trial proposal. In an additional exploratory analysis, the study aimed to investigate, whether problems in ToM and ER influence the quality of the therapeutic relationship (hypothesis 3). Further, we assessed in an additional exploratory analysis, whether positive ToM and ER performance moderate the association between delusions and the quality of the therapeutic relationship (hypothesis 4).

## Methods

### Subjects

Subjects were 185 patients with psychosis and 48 healthy controls from the “Cognitive behavioural therapy for persistent positive symptoms (CBTp) in psychotic disorders” Trial [32] (ISRCTN29242879), a multi-centred randomized controlled trial investigating the efficacy of CBT for patients with psychosis in comparison to supportive therapy.

Inclusion criteria were a diagnosis of a psychotic disorder (schizophrenia (*n* = 147), schizophreniform disorder (*n* = 1), schizoaffective disorder (*n* = 25) or delusional disorder (*n* = 12)) assessed with the Structured Clinical Interview for DSM-IV (SCID [33]).

Further inclusion criteria were persistent positive symptoms for at least the last three months and a minimum score of four in the item P1 (delusions in general: *n* = 162) and/or in the item P3 (hallucinations: *n* = 79; both: *n* = 56) of the Positive and Negative Syndrome Scale (PANSS [34]), age between 18 and 59, adequate language fluency and a verbal intelligence quotient > 80 in the German IQ test Mehrfachwahl-Wortschatztest (MWT-B [35]. Exclusion criteria for healthy controls were mental disorders in their lifetime as assessed with the Structured Clinical Interview for DSM-IV (SCID [33]).

Patients were recruited from six different psychiatric centers (Bonn, Cologne, Duesseldorf, Duisburg, Frankfurt am Main, Tuebingen, Germany); healthy controls were recruited in all six centers via public advertisement and matched with regard to age, gender, and education to the first 48 patients that were already recruited.

From the initial study sample (*n* = 330), a small number of patients dropped out before they were asked to participate (*n* = 9) or refused to participate in the additional assessment (*n* = 22), had problems with their eyesight (*n* = 3), with German language (*n* = 4), with test instructions (*n* = 14) or decided to participate in a nested fMRI study instead (*n* = 93). There were no statistically significant differences between the patients (*n* = 185) who performed in ToM and ER in the present study and patients who participated in the fMRI paradigm and between patients who refused to be tested and patients who endorsed testing with regard to sociodemographic and clinical variables (all *p* > .10).

All participants were informed about the assessment and gave written informed consent. In case of a legal guardian, the patients and the guardian were informed about the assessment and both the patient and the guardian gave written informed consent. The ethics committees at the six centres’ medical faculties approved the study. The randomized-controlled trial and the secondary analysis presented here adhere to Consort criteria.

From the total patient sample, a smaller number of patients participated in at least three therapy sessions and these patients were included in analyses regarding the association between ToM, ER, delusions and the therapeutic relationship (*n* = 174, CBT: *n* = 90, ST: *n* = 84).

### Measures

*Theory of Mind (ToM)* performance was assessed using a cartoon task paradigm [36, 37] that presented excellent test-retest reliability with regard to activation of key ToM areas in previous studies [38] Participants were asked to view 14 comic strips presented on a computer screen in pseudo-randomized order. Each comic strip included two phases: in Phase I, three pictures (3 s each) depicting an unfolding story plot were shown sequentially. In Phase II, two answer pictures were presented simultaneously (26 s), and participants were asked to choose the picture showing the logical ending of the story. The comic strips presented a social interaction between two protagonists (e.g. a person asks a second person for a glass of water). In order to solve the task, participants were required to infer the intentions of the characters correctly. The sum scores of correct answers were used as measure for *ToM* (range between 0 and 14).

*Emotion Recognition (ER)* was assessed with a Pictures of Facial Affect test (PFA). 28 faces (10 photographs selected from Ekman and Friesen’s pictures of facial affect [39] and 18 photographs from a comparable set of pictures [40, 41]) served as stimuli depicting four basic emotions (fear, anger, disgust and sadness). Each emotion was displayed by seven different faces showing each emotion once. Faces were presented sequentially in randomized order and participants were asked to select the most applicable emotion from a multiple-choice list. The PFA sum score of correct answers was used as measure of *ER* (ER total score; range between 0 and 28). In addition, scores of the four negative emotions were also used (ER fear, ER anger, ER sadness and ER disgust; ranges between 0 and 7).

The *Positive and Negative Syndrome Scale* (PANSS [34]) is a semi-structured interview assessing 30 symptoms divided into three scales (positive scale, negative scale, general psychopathology scale, ranges between 7 and 14 for the positive and negative scale and between 16 and 30 for the general psychopathology scale) using a 7-point Likert scale. PANSS ratings were performed by trained raters who received ten training sessions in all items. The inter-rater reliability (ICC, corr. R^2^) was satisfactory to high (between .86 and .92). The item P1 (general delusions) was used as assessment of general delusions and the item P6 as assessments of delusions of persecution (range of both items between 1 and 7).

The *Psychotic Symptom Rating Scale (PSYRATS* [42]*)* is a semi-structured interview with six items assessing different dimensions of delusional beliefs such as amount of preoccupation, duration of preoccupation, conviction, disruption of daily life, amount of distress and intensity of distress on a 5-point-Likert scale (scores between 0 and 4). Inter-rater-reliability and validity were high in a sample of patients with psychosis [42]. Based on results of a factor analysis [43], the items are summed up to the subscale *PSYRATS delusional frequency* (sum of the subscales amount of preoccupation, duration of preoccupation, conviction and disruption of daily life, range between 0 and 16) and *PSYRATS delusional distress* (sum of the subscales amount of distress and intensity of distress, range between 0 and 8).

*The quality of the therapeutic relationship* was assessed using the short versions of the Patient Session Questionnaire (PSQ) and the Therapist Session Questionnaire (TSQ) derived from the German Berner Therapist- and Patient- Session Questionnaire [Berner Patienten- und Therapeutenstundenbogen 2000]. Internal consistency (Cronbach’s alpha) was found to be good and effect sizes of correlations between quality of the therapeutic relationship viewed by the patient and therapist were generally large [44]. Both therapists and patients answered the questionnaires after the first five sessions of either CBT or ST. In order to reduce socially more desirable answers, patients answered the questionnaire after every session and put their ratings in a closed box.

From the PSQ, the *quality of the therapeutic alliance viewed by the patient* subscale (PSQ therapy alliance) was used that consists of the sum of three items that were answered by patients after a therapeutic session on a 7-point-Likert scale (scores between − 3 and + 3) and reflect the quality of the therapeutic relationship viewed by the patient (“The therapist and I understand each other”, “Today I felt at ease with the therapist”, “I think the therapist is really interested in my well-being”). In addition, from the TSQ, the 3-item subscale TSQ *therapist alliance* was used that measures the mean score of the *quality of the therapeutic relationship viewed by the therapist*. Items are answered on a 6-point-Likert scale (“The patient and I understand each other.” “Today I felt at ease with the patient.” “The patient and I work on joint goals.”; scores between − 3 and + 3). The mean score over the first five sessions was used (range between − 3 and + 3).

In addition, we used one item in the TSQ covering *interpersonal difficulties* viewed by the therapist (TSQ interactional difficulties: “I believe this patient is difficult in terms of his/her interaction”) that was answered on a 7-point Likert scale (scores between − 3 and + 3). Again, mean scores of the first five sessions were used (range between − 3 and + 3).

### Statistical analysis

First, we used Fisher’s exact tests, *Chi*^*2*^ tests and *t*-tests in order to compare patients with schizophrenia and controls in socio-demographic, clinical variables and ToM / ER problems (hypothesis 1). Exploration of the raw data showed ceiling effects for ToM and ER (total score and scores of the specific four negative emotions) as defined by Uttl [45] Thus, due to the skewedness of the data, we used an arcus-sinus-transformation as recommended by [46] that allows application of parametric statistics.

Hypothesis 2 (associations between ToM, ER and delusions) was assessed using Pearson’s bivariate correlations between ToM, ER (ER total score, ER fear, ER anger, ER sadness and ER disgust) and delusions in general (PANSS item P1), delusions of persecution (PANSS item P6) and the two PSYRATS subscales delusional frequency and delusional distress. Spearman rank correlations were used to examine hypothesis 3 (associations between ToM, ER total score, ER fear, ER anger, ER sadness and ER disgust and the therapeutic relationship), as PSQ and TSQ subscales were not normally distributed.

A series of hierarchical regression analyses were performed to assess whether ToM and ER problems moderate the association between delusions and the quality of the therapeutic relationship (hypothesis 4). All measures that were bivariately associated with the quality of the therapeutic relationship (PSQ therapy alliance / TSQ therapist alliance / TSQ interactional difficulties), were mean-centered and then included as predictors in the first block of the hierarchical regression analysis and their interaction term in the second block. General symptom severity (PANSS total score) was included as covariate.

Significant moderating relationships were analysed using the Johnson-Neyman technique included in the PROCESS macro [47], a follow-up method for regressions containing interaction coefficients that enables to identify over what range of the moderator a predictor has significant versus non-significant effects on the outcome measure [48].

## Results

Table 1 shows socio-demographic and clinical data of patients with psychosis and healthy controls. There were no statistically significant differences between both groups in terms of age or gender. Compared to controls, patients showed significantly lower verbal intelligence scores (MWT-B), but associations between ToM, ER and verbal intelligence were small and not statistically significant and no statistical adjustment for verbal intelligence was performed.

**Table 1:** Sociodemographic and clinical variables of patients with psychosis and healthy controls

	*n*(patients/ Controls)	Patients with psychosis (*n* = 185)	Healthy controls (*n* = 48)	Test statistics
		*n* (%) / M (SD)	*n* (%)/ M (SD)	
Age (ys.)	185/48	38.62 (9.78)	35.69 (9.44)	*t*(231) = 1.865, *p* = .063
Gender (fem.)	185/48	107 (57.84%)	20 (41.68%)	^a^*p* = 1.000
School education:	185/48			*X*^2^(4)=8.331, *p* = .080
13 years		97 (52.43%)	24 (50%)	
10 years		56 (30.27%)	22 (45.83%)	
9 years		31 (16.76%)	2 (4.17%)	
Less than 9 years		1 (0.54%)	–	
Verbal intelligence (MWT-B) ^b^	176 / 46 ^b^	107.48 (14.82)	114.52 ((15.36)	*t*(222) = −2.85, *p* = .005
Duration of illness	185	15.36 (9.63)		
Theory of Mind (ToM) ^c^	185/39 ^c^	12.56 (1.74)	13.31 (1.00)	*t*(222) = −2.711, *p* = .007
ER total score	185/45^d^	23.31 (3.23)	24.8 (2.06)	*t*(228) = −2.954, *p* = .003
ER fear	185/45^d^	5.90 (1.43)	6.22 (0.90)	*t*(228) = − 1.456, *p* = .147
ER anger	185/45^d^	6.05 (1.19)	6.69 (0.56)	*t*(228) = −3.512, *p* = .001
ER sadness	185/45^d^	6.28 (1.15)	6.33 (1.09)	*t*(228) = −.275, *p* = .783
ER disgust	185/45^d^	5.09 (1.28)	5.56 (1.16)	*t*(228) = −2.247, *p* = .026
PANSS positive scale	185	17.71 (3.79)		
PANSS negative scale	185	13.57 (4.17)		
PANSS general psychopathology scale	185	32.62 (7.27)		
PANSS P1 delusions in general	185	4.44 (1.02)		
PANSS P6 delusions of persecution	185	3.37 (1.57)		
PSYRATS delusional frequency	185	8.76 (3.56)		
PSYRATS delusional distress	185	5.1 (2.46)		
TSQ therapy alliance (therapist)	174 ^d^	1.09 (.65		
TSQ interactional difficulties (therapist)	174 ^d^	−.50 (1.23)		
PSQ therapy alliance (patient)sessions 1–5	174 ^d^	1.72 (.62)		

Notes: ^a^ Fishers Exact Test; ^b^*n* = 9 patients did not perform in the MWT-B due to language problems and two controls due to organisational problems, ^c^*n* = 9 controls did not participate at the ToM assessment, ^d^*n* = 3 controls did not participate at the ER assessment; ER: Emotion recognition; PANSS: Positive and Negative Syndrom Scale; PSYRATS: Psychotic Symptoms Rating Scale; TSQ therapy alliance = Therapist session questionnaire = quality of therapeutic relationship viewed by the therapist (mean score sessions1–5); TSQ interactional difficulties = Therapist Session Questionnaire rating of interactional difficulties rated by the therapist (mean score sessions 1–5), PSQ therapy alliance = Patient Session Questionnaire rating of the quality of the therapeutic relationship rated by the patient (mean score sessions 1–5); ^d^ TSQs were filled by 13 different therapists and 174 patients

### Group differences in ToM and ER (hypothesis 1: pre-specified analysis)

**Fig. 1:**
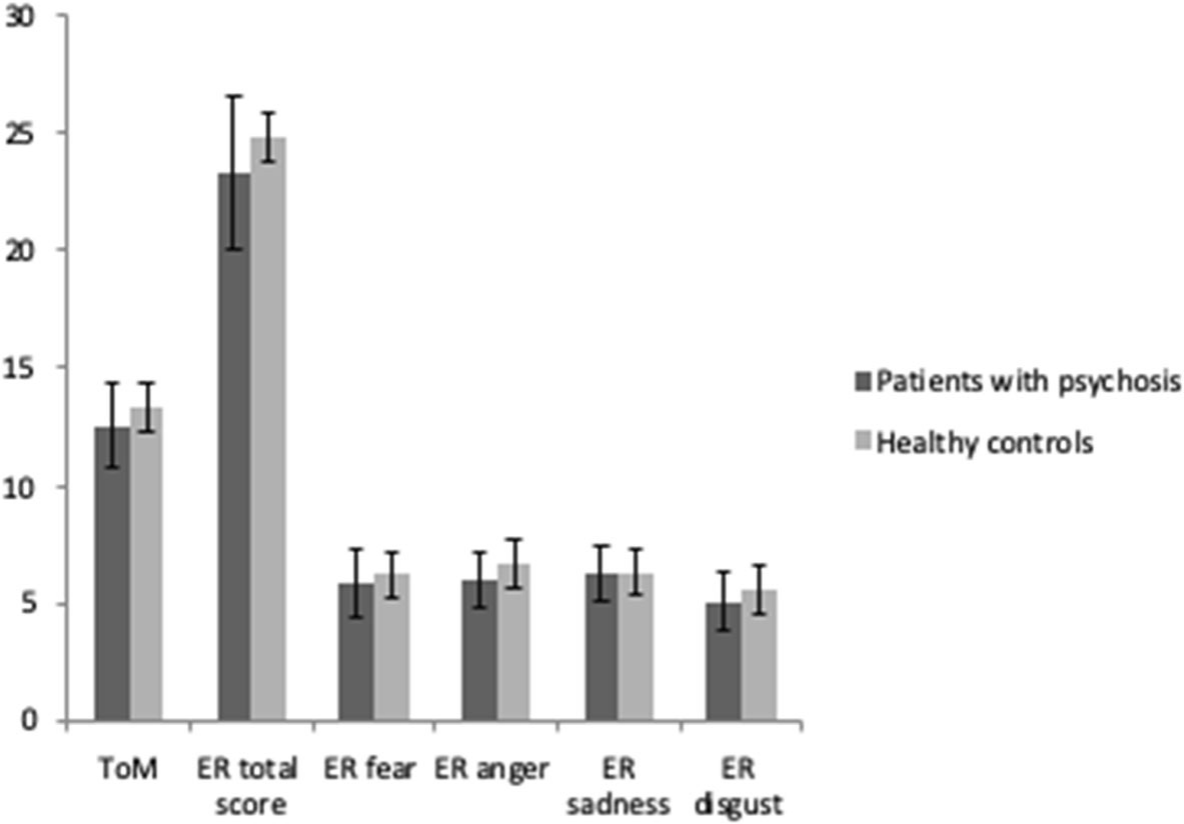
ToM and Emotion Recognition in patients with psychosis and healthy controls. Notes: ToM = Theory of Mind, ER = Emotion Recognition; Patients with psychosis: *n* = 185; Healthy controls: Theory of Mind: *n* = 42; Emotion Recognition: *n* = 39, error bars display the standard deviation

With regard to hypothesis 1, results revealed that patients with psychosis presented more pronounced deficits in both ToM and ER (descriptive scores are depicted in Fig. 1). With regard to ER, patients were more impaired than controls in the total ER score and in recognizing the emotions anger and disgust, whereas there were no statistically significant differences between both groups with regard to recognizing the emotions fear and sadness.

### Associations between ToM, ER and delusions (hypothesis 2: pre-specified analysis)

As depicted in Table 2, there was no statistically significant association between ToM problems and any measure of delusions. However, problems in ER were related with more pronounced PSYRATS delusional distress. Specifically, delusional distress (PSYRATS delusional distress) showed a statistically significant association with the ability to recognize the emotions fear and disgust (ER fear and ER disgust), whereas the two other specific ER scores (ER anger and ER distrust) were not associated with delusional distress. Further, neither the ER total score nor the four specific negative emotions scores were related to any other measure of delusions.

**Table 2:** Results of Pearson correlation analyses between Theory of Mind, emotion recognition and delusions in the patient sample *(n* = 185)

	ToM	ER total score	ER fear	ER anger	ER sadness	ER disgust
ER total score	.316**					
ER fear	.207**	.743**				
ER anger	.244**	.462**	.052			
ER sadness	.171*	.688**	.479**	.230		
ER disgust	.228**	.690**	.389**	.057	.356**	
PANSS P1 delusions in general	.096	.006	.014	.096	−.100	−.033
PANSS P6 delusions of persecution	.113	.021	−.012	.101	−.019	.048
PSYRATS delusional frequency	−.057	.068	.064	.070	−.028	.018
PSYRATS delusional distress	.020	.219**	.166*	.085	.124	.236**

Notes: *ER* Emotion recognition, *PANSS* Positive and Negative Syndrom Scale, *PSYRATS* Psychotic Symptoms Rating Scale, *: *p* < .05; **: *p* < .01

### Associations between ToM, ER, delusions and the quality of the therapeutic relationship (hypothesis 3: exploratory analysis)

Patients’ ratings of the quality of the therapeutic relationship (PSQ therapy alliance) were not related with ToM nor ER total score and ER subscales (see Table 3). In addition, there was no statistically significant association between PSQ therapy alliance and all other measures of delusions. Therapists’ ratings of the quality of the therapeutic relationship (TSQ therapy alliance) were related to ER total score and to all four ER subscales (ER fear, ER anger, ER sadness and ER disgust) and PSYRATS delusional frequency. TSQ interactional problems showed a statistically significant association with ER total score and PSYRATS delusional frequency. In addition, TSQ interactional problems were related with problems in recognizing the emotions fear and disgust (ER fear, ER disgust), whereas there were no statistically significant associations between TSQ interactional problems and problems in recognizing anger and sadness (ER anger and ER sadness).

**Table 3:** Results of Spearman correlation analyses between Theory of Mind, emotion recognition and the quality of the therapeutic relationship in the patient sample

	PSQ therapy alliance viewed by the patient (*n* = 174)	TSQ therapy alliance viewed by the therapist *(n* = 174)	TSQ interactional difficulties viewed by the therapist (*n* = 174)
PSQ therapy alliance	–	.240**	−.222**
TSQ therapist alliance	.240**	–	−.701**
TSQ interactional difficulties	−.222**	−.701**	–
ToM	−.014	.112	−.077
ER total score	−.039	.347**	−.278**
ER fear	−.128	.186*	−.154*
ER anger	.009	.170*	−.078
ER sadness	.079	.340**	−.268
ER disgust	.018	.262**	−.237**
PANSS P1 delusions in general	−.042	.040	.113
PANSS P6 delusions of persecution	−.002	.100	.162
PSYRATS delusional frequency	−.039	−.136*	.254**
PSYRATS delusional distress	−.053	.003	.139*

Notes: *ER* Emotion recognition, *PANSS* Positive and Negative Syndrom Scale, *PSYRATS* Psychotic Symptom Rating scale; *PSQ* therapy alliance Patient Session Questionnaire, *TSQ* therapy alliance Therapist session questionnaire; *: *p* < .05; **: *p* < .01

### Moderation analyses (hypothesis 4: exploratory analysis)

As can be seen in Table 4, including ER and PSYRATS delusional frequency in the first block of the hierarchical regression analysis predicting TSQ therapist alliance resulted in a statistically significant model, but solely ER was a statistically significant predictor of TSQ therapist alliance. Including the interaction term in the second block resulted in a statistically significant model and the interaction explained additional variance in TSQ therapy alliance, suggesting a statistically significant moderation effect.

**Table 4:** Hierarchical multiple regression analyses predicting the quality of the therapeutic alliance and interactional problems

Predictors	B	t	p	F / F _Change_	df	p	Adjusted R^2^ / R^2^_change_
Regression 1: TSQ therapist alliance ^1^							
Step 1 **				7.741	3168	< .0001	.106
PANSS total (covariate)	.011	.144	.886				
Emotion Recognition**	.339	4.643	< .0001				
PSYRATS delusional frequency	−.110	−1.399	.164				
Step 2 **				7.406	1167	.007	.037
PANSS total (covariate)	−.018	−.230	.818				
Emotion Recognition **	.288	3.881	< .0001				
PSYRATS delusional frequency	−.057	−.715	.475				
Interaction**:	.205	2.721	.007				
Regression 2: TSQ interactional problems ^1^							
Step 1 **				7.741	3168	< .0001	.106
PANSS total (covariate)**	.221	2.911	.004				
Emotion Recognition **	−.252	−3.565	<.0001				
PSYRATS delusional frequency*	.176	2.316	.022				
Step 2 **				7.406	1167	.007	.139
PANSS total (covariate)**	.244	3.207	.002				
Emotion Recognition**	−.213	−2.942	.004				
PSYRATS delusional frequency	−.135	1.749	.0982				
Interaction*	−.156	−2.125	.035				

Notes: *TSQ* Therapist Session questionnaire, *PANSS* Positive and Negative Syndrome Scale, *PSYRATS* Psychotic Symptom Rating Scale; ^1^: 174 patients participated at least three sessions and the quality of the therapeutic relationship was rated by their therapists (*n* = 13); *: *p* < .05; **: *p* < .01

The Johnson-Neyman technique revealed that ER was predictive of TSQ therapy alliance for participants with ER scores lower than 20.45% and above 79.65% (all *p <* 0.05). This suggests that in patients with scores in ER in percentiles between zero and 20.45, ER problems attenuated the negative influence of delusional frequency on TSQ therapy alliance and in patients with ER scores in percentiles above 79.65%, good ER ability protected them from the negative impact of delusional frequency on TSQ therapy alliance.

Results of the second hierarchical regression analysis revealed a statistically significant model including ER and PSYRATS delusional frequency as predictors of TSQ interactional problems, but solely ER independently predicted interactional problems, whereas PSYRATS delusional frequency was not independently predictive of interactional problems. Including the interaction term in the second block resulted in a statistically significant model that explained a significant additional amount of variance in TSQ interactional problems and suggested a moderation effect.

The Johnson-Neyman technique revealed that ER moderated the negative influence of delusional frequency on TSQ interactional problems only in patients with ER scores (percentiles) between zero and 54.07% (all *p <* 0.05) suggesting that ER problems attenuated the negative impact of delusional frequency on TSQ interactional problems only in these patients. In persons with higher ratings, the moderation effect was not present.

## Discussion

In comparison to controls, patients with psychosis presented problems in both ToM and emotion recognition (ER). Nevertheless, solely problems in ER were related to delusional distress, but not related to other measures of delusions. Problems in ER also had an impact on the quality of the therapeutic alliance and interactional problems viewed by the therapist. Finally, good ER ability reduced the negative impact of delusional frequency on both the quality of the therapeutic relationship and interactional problems during CBT/ST.

The present study is the first study reporting that problems in ER are associated with delusional distress, whereas there were no further associations with other measures of delusions. In particular, problems in recognizing the negative emotions fear and disgust were related with more pronounced delusional distress, whereas problems in recognizing the emotions anger and sadness were not related to delusional distress. Our results suggest that problems in ER (especially problems in recognizing fear and disgust) might not be directly related to the presence of delusions, but enhance the distress associated with them, in line with experimental designs suggesting that problems in ER are more pronounced in stressful situations in patients with psychosis [49]. It is possible that in stressful situations, patients with psychosis are less able to interpret social cues correctly and perform more errors in recognizing emotions and in inferring emotional states of other persons [50, 51] and this might lead to interactional problems and reduced social functioning [23]. Thus, it might be useful to further investigate using longitudinal assessments whether problems in ER - although not directly associated with the intensity of delusions - might increase delusional distress. In addressing this question, it is highly important to measure ER problems with regard to specific negative emotions, as our results demonstrate that problems in recognizing the emotions fear and disgust are related to delusional distress, whereas problems in recognizing anger and sadness were not related to more pronounced delusional distress. If longitudinal associations between problems in ER and specific negative emotions and delusional distress are further established, ER problems should then be included as one of the cognitive factors involved in the development and maintenance of delusional distress in theoretical models.

The fact that we did not find an association between ToM problems and any measure of delusions nor delusional frequency / distress is in line with findings of two meta-analyses [14, 18] and several other studies that did not report an association between ToM and delusions [8, 52, 53], whereas negative symptoms and symptoms of disorganisation are more constantly associated with ToM problems [14]. One explanation might be that ToM problems are less pronounced in patients with delusions in comparison to patients with negative or disorganized symptoms (e.g. [54, 55], see Spronghorst et al. for a review on the literature of subgroup comparisons [56]) and thus, harder to assess using typical ToM paradigms that sometimes lack ecological validity [57]. Interestingly, all studies that used more ecologically valid ToM assessments such as movies of social situations (e.g. the Movie of Assessment of Social Cognition (MASC) [58] or the Movie Task of social situations [20]), found associations between ToM problems and more pronounced general and persecutory delusions in patients with psychosis [59].

An additional limitation of current ToM paradigms is the fact that they often measure ToM in a wright-or-wrong format and thus investigate solely reduced ToM abilities/undermentalizing in patients with psychosis, whereas Abu-Akel [60, 61] suggested that patients with delusion rather present problems in overmentalizing / “hypermentalizing” mental states of other persons, defined as Hyper-ToM [58, 62]. First studies addressed the question of associations between Hyper-ToM and delusions in children with psychotic experiences and normal controls [63, 64] and patients with psychosis [58] and found evidence of an association. Thus, Hyper-ToM rather than undermentalizing might play an important role in the formation and maintenance of delusions and should be investigated in future studies.

Concluding, future studies that address the question of associations between ToM problems and delusions might be well-advised to use tasks with more pronounced ecological validity, for example, ToM assessment using videos, virtual reality (Virtual Assessment of Mentalising Ability (VAMA) [65] or investigating ToM problems in real-life using the experience sampling method [66]. Nevertheless, if there are still no associations between problems in Hyper-ToM and delusions, theoretical models correctly excluded ToM as one of the important cognitive factors involved in the formation and maintenance of delusions or positive symptoms [12, 13].

Our study is the first to report that therapists who treated patients with problems in ER (especially problems in recognizing the emotions disgust and fear) perceived more pronounced interactional problems in these patients. In addition, therapists who treated patients with problems in ER (especially in recognizing the emotions fear, anger, sadness and disgust) rated the quality of the therapeutic relationship more negatively. Our findings are partly in line with the study of Jung and colleagues [29] who reported an association between patients’ ratings on the quality of the therapeutic alliance and ToM problems, but no associations between therapists’ ratings and ToM problems, but the size of our study sample enabled us to detect associations of medium and small effect size. Nevertheless, it has to be taken into account, that our results were not pre-registered, but obtained in an exploratory analysis of a randomized-controlled therapy trial, thus, careful replication of our results should be performed, especially in light of the current replication crisis in psychology (see [67] for a review)). If our results are successfully replicated and patients’ problems in ER influence the relationship with an empathetic and highly skilled therapist, it can be assumed that their problems in ER also have a negative impact on other social interactions in their daily life, as suggested by several other studies that directly addressed this question [21, 22].

In addition, we could provide evidence for the clinically important negative impact of delusional frequency on both the therapeutic relationship and interactional problems viewed by the therapist. Again, it is important to note that these results were obtained in an exploratory analysis and thus are in need of careful replication. If our findings are replicated in longitudinal pre-registered assessments, they suggest that delusional frequency negatively affects social interactions (the therapeutic interactions) and thus may also partly contribute to the association between delusions and lower social functioning [68, 69], negative family atmosphere [70], more pronounced loneliness [71, 72] and social exclusion [73, 74].

Further, preserved ER abilities might protect patients from the negative influence of their delusions on the quality of the therapeutic relationship and interactional problems, as a statistically significant moderation effect occurred. Interestingly, the interaction was most pronounced in patients with severe ER problems: in this subgroup, problems in ER had a specific negative influence on both the therapeutic relationship and interactional problems. Our results are partly supported by a second study that addressed the impact of ToM on the association between delusions and self-rated social functioning [31]. Their results also suggest that preserved ToM abilities moderated the relationship between persecutory delusions and self-rated social functioning [31]. The fact that we did not find a similar moderation effect between ToM and the quality of the therapeutic relationship could be explained by different ToM assessments: the study used the Hinting task [54]) that is based on verbal descriptions of social situations, we used a picture sequencing test based on comics. To some degree, our findings expand on their results, as we used therapists’ ratings of the therapeutic alliance as a direct measure of social functioning instead of self-ratings. Again, if our exploratory findings can be replicated and positive ER skills are a protecting factor against the negative influence of delusions on the therapeutic relationship, patients’ ER abilities might also influence the effectiveness of CBT for psychosis, as a positive therapeutic relationship is closely related to the effectiveness of CBT [28, 75, 76]. Interestingly, one study indeed found general ToM abilities (including ER) to moderate change in positive symptoms in CBT [77]. Thus, interventions that improve ER and ToM abilities might be beneficial in order to improve the therapeutic relationship and, further, the effectiveness of CBT.

ER and ToM problems in patients with psychosis can also be viewed in a broader perspective as parts of patients’ more general problems in their metacognitive capacities. Metacognition has been defined as “cognition about cognition” by Flavell [78] and also discussed as an important part of social cognition [79]. Both ToM and ER are important parts of metacognition, in combination with *self-reflectivity* (comprehension of one’s own mental state), *decentration* (the ability to from a complex representation of the world) and *mastery,* the ability to use information of one’s own and other mental states in respond to and to solve social and psychological problems [80].

In comparison to controls, patients with psychosis were found to present problems in almost all parts of metacognition (see Lysaker et al. [80] for a review on metacognition in schizophrenia). Metacognitive abilities in patients with psychosis are closely linked to a positive therapeutic relationship viewed by patients in CBT (mastery [81]) and also with positive outcome in Cognitive Remediation therapy (learning potential: [82]). Thus, as ER is an important part of metacognition, it is plausible that we also found a link between problems in ER and a less favourable therapeutic relationship and interactional problems viewed by the therapist, as ER can be viewed as one part of metacognitive mastery that was also found to be linked to a positive therapeutic relationship [81]. Thus, the association between problems in ER and a less favourable therapeutic relationship could be moderated by general metacognitive deficits in patients with psychosis. Therapists might perceive these deficits during the first therapeutic sessions and these problems might influence the therapeutic relationship.

For example, patients with deficits in ER and metacognition might present problems in their metacognitive self-reflection that could become visible in diagnostic sessions, as they might not be able to talk about their individual thoughts and emotions in specific situations. They also might present problems in understanding the basic cognitive model that consists of relations between individual perceptions, thoughts, emotions and behaviour [83] due to their problems in self-reflection. Further, patients might also present problems in *decentration* and thus might not be able to form a complex representation of the world that is important in therapy in order to solve personal and interpersonal problems, e.g. due to their well-known jumping-to-conclusions-bias [84]. Finally, patients’ level of *mastery* in using their information on mental states in order to solve real-world problems might also be reduced.

Concluding, it is possible that the association between poor emotion recognition and the therapeutic relationship/interactional problems viewed by the therapist can be explained by patients’ metacognitive problems. In addition, it is plausible that not only ER abilities but also a positive metacognitive performance might moderate the influence of delusional frequency on the therapeutic relationship and thus, might also be helpful for patients with psychosis in their general social life, as suggested by a study that found metacognitive capacities to mediate the negative influence of neurocognitive deficits on social functioning in patients with psychosis [85]. Thus, future studies will be well-advised to address all aspects of metacognition in patients with psychosis and their influence on the therapeutic relationship.

Our results suggest for therapists of patients with psychosis to take patients’ potential ER problems (and their metacognitive deficits) into account in CBT for psychosis. First, it could be useful to assess patients with regard to their ER abilities before start of therapy. Second, if patients present ER problems, it is important for therapists to make a special effort to improve the therapeutic relationship with these patients. Third, it could be helpful to train ER (and metacognition) in patients with psychosis using specialized interventions from several social cognition trainings in the framework of Cognitive Remediation [86]: the Social Cognition and Interaction Training [87] and the Metacognitive Training (Moritz and Woodward [88]) aim on improving both ToM and ER, whereas the Training of Affect Recognition [41] aims more closely on ER. In general, these trainings were able to enhance both ToM and ER abilities [87, 89–91] and their general positive effect on social functioning is large [92, 93]. It is also possible, that an integration in or a combination of these trainings with CBT in order to improve ER might be beneficial.

### Strength and limitations

Strength*s* of the present study include the large sample of patients with psychosis and the detailed assessment of different dimensions of delusions. An additional strength is the longitudinal assessment of the quality of the therapeutic relationship over five sessions.

In interpreting our findings, it should be mentioned that all associations between ToM, ER, delusions and the therapeutic relationship were of small effect size according to Cohen [94]. In addition, it should be noted that solely two of the four scientific hypotheses were pre-specified, whereas all associations between ER, ToM, delusions and the therapeutic relationship were of exploratory nature. Thus, the question of associations between ToM, ER and the therapeutic relationship and the moderation effect require an additional careful replication study.

In addition, while our moderation model and the mode of assessment (ER and delusions were assessed before the start of therapy) suggest a causal association between problems in ER and delusional frequency and also implicate that positive ER abilities moderate the association between delusional frequency and the therapeutic relationship, we cannot rule out the possibility that a low quality of the therapeutic relationship and interactional problems were influenced by other factors, e.g. common therapeutic factors such as therapists’ empathy, expertness, attractiveness and trustworthiness [95, 96] and thus, it is also possible that an unfavourable therapeutic relationship might lead to more pronounced delusions in patients with psychosis. Thus, future studies should focus on symptom change, ER and the therapeutic relationship using multiple assessments in order to address the question whether more pronounced ER problems cause more pronounced delusional frequency/distress and a less favourable therapeutic relationship (or vice versa).

Finally, it should be mentioned that the patients in the present study were patients interested in participating in a therapy trial who might present better general cognitive and social functioning and less pronounced problems in their social cognition. Nevertheless, as the moderation effect occurred predominantly in patients with lower ER skills, a potential selection bias might not influence the generalisation of our exploratory results.

## Conclusions

Several important conclusions can be drawn from our results. First, problems in ER were related to enhanced delusional distress in patients with psychosis. In addition, patients’ deficits in ER had a negative impact on the quality of the relationship viewed by their therapists. Nevertheless, positive ER in patients with psychosis moderated (reduced) the negative impact of delusions on the therapeutic relationship. Thus, improving ER might be a way to improve the quality of the therapeutic relationship and potentially the effectiveness of CBT or other interventions for patients with psychosis.

## Data Availability

The dataset is available from the corresponding author on reasonable request.

## Abbreviavions

CBT: Cognitive Behavior Therapy.
CBTp: Cognitive Behavior Therapy for positive symptoms of psychosis.
DSM-IV: Diagnostic and Statistical Manual of Mental Disorders, 4th Edition.
ER: Emotion Regulation.
fMRI: functional Magnetic Resonance Imaging.
MWT-B: Mehrfachwahlwortschatztest (German verbal intelligence test).
PANSS: Positive and Negative Syndrome Scale.
PFA: Pictures of Facial Affect Test.
PSQ: Patient Session Questionnaire.
PSYRATS: Psychotic Symptoms Rating Scale.
ST: Suppportive Therapy.
ToM: Theory of Mind.
TSQ: Therapist Session Questionnaire.
